# Rifampin-Associated Flu-Like Syndrome in a Patient Undergoing Treatment for a Device-Related Infection

**DOI:** 10.7759/cureus.12336

**Published:** 2020-12-28

**Authors:** Loreto L Calaquian, Asha De

**Affiliations:** 1 Internal Medicine, Brian D. Allgood Army Community Hospital, Seoul, KOR; 2 Internal Medicine, San Antonio Military Medical Center, San Antonio, USA

**Keywords:** rifampin, rifampicin, side-effects, device-related infection

## Abstract

Rifampin (or rifampicin) has found extensive use for the treatment of a variety of infectious illnesses, particularly tuberculosis and device-related infections. We describe the development of a flu-like syndrome in a patient undergoing extended antimicrobial therapy with rifampin for discitis and an associated device-related infection, which promptly resolved with discontinuation of rifampin. While the flu-like syndrome has been documented in prior literature covering the treatment of tuberculosis (where the dose regimen tends to be intermittent) there is less evidence for its occurrence in the treatment of device-related infections (which tend to be dosed daily).

## Introduction

Rifampin is a versatile drug classically known as the first line of therapy in the treatment of Mycobacterium tuberculosis, but it has also come to be a favored agent in the treatment of device-related infections, particularly gram-positive bacterial species [1]. The breadth of our clinical experience with rifampin has been drawn from data obtained from patients undergoing therapy for tuberculosis, including our understanding of the adverse side effects of rifampin and the frequency of their occurrence. As with other drugs, the dosing of rifampin varies significantly depending on the indication for which it is prescribed which may lead to differences in the clinical presentations of side effects [1,2]. We present a case of a patient who developed adverse effects while undergoing therapy with rifampin for a device-related infection, with notable differences from previously described cases.

## Case presentation

**Figure 1:**
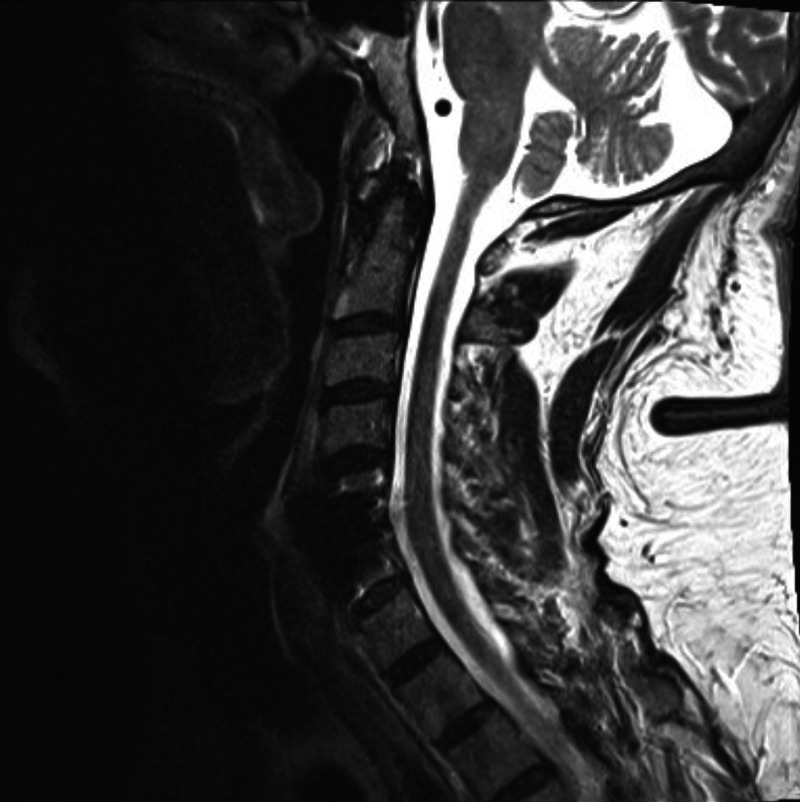
Cervical spine MRI obtained during admission

The patient is a 66-year-old male with a past medical history of depression, osteoarthritis, post-traumatic stress disorder (concurrently taking duloxetine 60mg daily, mirtazapine 30mg nightly, and aripiprazole 2mg daily), benign prostatic hypertrophy (on alfuzosin 10mg daily), and obstructive sleep apnea who following an analgesic spinal nerve block developed C5-C6 discitis with an associated epidural abscess. The patient was treated with debridement, discectomy, and subsequent fusion of C5 and C6 vertebrae. Wound cultures obtained grew Staphylococcus epidermidis and he was subsequently placed on a continuous cefazolin infusion and twice-daily oral rifampin (300 mg) as part of his outpatient antimicrobial therapy. He tolerated the regimen with no adverse effects for two weeks, with regular follow-ups and outpatient lab monitoring. On week three of therapy, he had an abrupt onset of intermittent fevers (to a maximum of 102F) and malaise, and subsequently on week four of therapy, developed polyarthralgia involving the bilateral knees, ankles, and left wrist. He denied any swelling, erythema, or warmth of the joints, and the review of systems was otherwise negative. Following an episode of transient hypotension, he was admitted to the hospital on week four of therapy. Out of concern for possible treatment failure, his antibiotic regimen was initially broadened to include vancomycin and ceftriaxone with the continuation of his outpatient twice daily rifampin. Physical exam noted no evidence of pain on palpation of joints, swelling, or erythema. Lab workup was notable for thrombocytopenia (83000), and elevated inflammatory markers that were up trending from recent outpatient labs (erythrocyte sedimentation rate peaking to 39 on admission from 17 and C-reactive protein peaking to 9.3 from 1.8 during the month prior to admission). He had normal white blood cell count, hemoglobin, renal and hepatic panels, and negative blood cultures. Repeat magnetic resonance imaging studies demonstrated no evidence of a new inflammation or infection in the spine (Figure 1) and imaging of the affected joints was notable only for osteoarthritis. Despite the broadening of antibiotics for 48 hours after admission, the patient had little change in clinical symptoms and continued to have persistent fevers, elevated inflammatory markers, and arthralgia. Lacking evidence for ongoing infection, suspicion fell on a possible drug reaction, and antibiotic therapy was entirely discontinued. Upon discontinuation of rifampin, the patient experienced a rapid improvement in all symptoms with marked normalization of vital sign derangements (including defervescence within 48 hours). The patient was subsequently switched to oral minocycline and completed the remainder of his antimicrobial therapy without further recurrence of symptoms.

## Discussion

The development of rifampin began in the mid-20th century from compounds isolated from bacteria now known as Amycolatopsis rifamycinica. From these, the compound rifamycin B was found to have relatively low antimicrobial activity (though sufficiently active in animal models), but of greater importance, an encouragingly low level of toxicity in vivo. The eventual down-stream byproduct of this compound would lead to the development of rifamycin SV, which would find use as an intravenous antimicrobial drug primarily in the treatment of gram-positive bacterial infections. The ongoing search for a more effective, and more importantly, an oral version of this class of medications would lead to further research to identify the specific molecular components responsible for therapeutic activity (several hydroxyl groups working in unison with another pair of polar groups), and other molecular subunits which could feasibly be substituted for to achieve a more ideal drug compound. The development of rifampin (a hydrazone derivative of rifamycin SV) would be the result of this work, and would quickly become accepted by the Food Drug Administration for use in the treatment of Mycobacterium tuberculosis, though early studies would quickly begin identifying its utility in a variety of other types of infections [2]. 

Rifampin has additionally proven itself to be an essential component of therapy in the treatment of prosthetic joint infections and device-related infections. As an oral medication with excellent efficacy against gram-positive organisms even in the presence of biofilm, rifampin rapidly emerged as the keystone drug in the treatment of similarly stubborn prosthetic related infections (though is often prescribed alongside other antimicrobial drugs due to concerns for resistance) [3,4]. Patients diagnosed with device-related infections will initiate induction antimicrobial therapy with a combination of drugs including rifampin and continue on this regimen for an extended course of curative therapy, though rifampin will likely be discontinued after several weeks if chronic suppressive therapy is required.

In the treatment of tuberculosis, rifampin rapidly augmented or supplanted already existing therapeutic regimens, all of which require longer, extended courses needed to ensure complete eradication of stubborn mycobacterial infections [5]. It is important to note that much of our understanding of the adverse effects of rifampin is derived from observations of these side effects suffered by patients on therapy with rifampin for tuberculosis. One such side effect is a flu-like syndrome, characterized by fevers, chills, malaise, and arthralgia. The symptoms, which tend to occur late into the patients’ treatment course, manifest several hours after drug administration, and can last for several hours afterward before abating. The reaction (like many associated with rifampin) appears to be dose-dependent, but the exact pathophysiology of the reaction has not been elucidated with absolute certainty [6-8]. There is some suspicion that this side effect may be related to anti-rifampin antibodies, but there is no clear association between symptoms and elevated levels of anti-rifampin antibodies. Similarly, there is little evidence that symptoms are related to an IgE-mediated process [7,9-12].

Following several weeks of therapy with a daily-dose rifampin-combination regimen, our patient developed fevers, chills, malaise, and diffuse pain that led us to suspect an infectious etiology, either new or refractory to therapy. Initial management with broadened empiric antibiotics did little to improve symptoms, and work-up for infectious etiologies returned negative, but discontinuation of rifampin resulted in rapid resolution of symptoms and prompt clinical improvement. The constellation of symptoms, time course of complaints, and prompt resolution following discontinuation of the offending agent mirror known side effects of rifampin, but there were several aspects to the case which did not match prior reports, and bear some discussion. 

The most significant difference in our case is that our patient developed symptoms with daily dosing of rifampin. Observational studies have noted in multiple cohorts that the reaction is mostly associated with regimens which include intermittent (either weekly, twice weekly, or every other day) dosing of rifampin (almost never with daily regimens), and resolves with either discontinuation of rifampin or switching to a daily-dose regimen [5-7,9-11,13]. The highest incidence of side effects seems to occur with once-weekly dosing of rifampin with higher doses [5]. Our patient, while admittedly not undergoing directly observed therapy as he would have if undergoing therapy for tuberculosis, had frequent outpatient follow-ups, was undergoing care at least several times weekly with a home health medical service, and reported absolute adherence to the daily dosing regimen he was prescribed for treatment of his discitis. Therefore we do not suspect that missed doses of medication were a contributing factor to his symptoms. 

Similarly, the rapidity with which our patient developed his symptoms (within three weeks of medication initiation) is discordant with the pattern of symptom onset identified in prior reviews of rifampin side effects. As noted above, prior studies note the development of flu-like symptoms in patients who were well into their treatment course. In particular, we found no noted episodes of flu-like symptoms prior to three months of continuous therapy, with multiple cases of patients manifesting similar episodes following periods as long as 15 months [5,9,11]. 

Another point of difference is the dosage at which our patient developed symptoms. Existing literature notes that patients found to have elevated serum levels of rifampin are more likely to report any adverse side effects with rifampin [6-8]. Though we did not order serum levels on the patient, we do not suspect that an elevated serum level of rifampin contributed to patient symptoms. His dose of rifampin (300mg twice daily for a total daily dose of 600mg) was somewhat higher than the daily dose described in prior literature (dosed for treatment of mycobacterium tuberculosis), though well within the normal dosing range for both tuberculosis and device-related infections [5]. Of note, our patient had no prior history of either hepatic or renal disease, with no suspicion that he would have had difficulty metabolizing or eliminating rifampin or its by-products. Besides the flu-like syndrome, the patient did not have any evidence (either reported symptoms or laboratory abnormalities) of other rifampin-associated adverse effects that would suggest elevated drug levels (i.e. acute renal failure, facial rash/flushing, anaphylaxis, acute hepatic injury, gastrointestinal distress, etc.) [5,9]. While obtaining rifampin serum levels or checking for the presence of anti-rifampin antibodies may have helped confirm the diagnosis, there is no particularly clear evidence that elevations in serum levels or the presence of antibodies would clearly be associated with symptoms.

## Conclusions

While undergoing extended antimicrobial therapy with twice-daily rifampin for a device-related infection our patient developed fevers, chills, malaise, and arthralgia all of which resolved following discontinuation of rifampin. Much of our knowledge about this medication and its adverse effects has been drawn from decades of experience using it for the treatment of mycobacterium tuberculosis, especially in regions where tuberculosis is endemic. In regions where tuberculosis is less common, the increasing use and familiarity with rifampin are driven by its indicated usage for the treatment of device-related infections. We owe a great debt of knowledge to the work of past clinicians. Based on their recorded experiences we can extrapolate data to inform our practice, but as in our patient, there can still be room for incongruity. The pathophysiology of many rifampin-associated adverse effects remains unclear at this time, and the other effects that may arise from the usage of rifampin in alternative roles remain to be discovered with further clinical experience and evaluation. 

## References

[1] Osmon DR, Berbari EF (2012). Diagnosis and management of prosthetic joint infection: clinical practice guidelines by the Infectious Diseases Society of America. Clin Infect Dis.

[2] Sensi P (1983). History of the development of rifampin. Rev Infect Dis.

[3] Widmer A, Gaechter A, Ochsner P, Zimmerli W (1992). Antimicrobial treatment of orthopedic implant-related infections with rifampin combinations. Clin Infect Dis.

[4] Zimmerli W, Sendi P (2018). Role of rifampin against staphylococcal biofilm infections in vitro, in animal models, and in orthopedic-device-related infections. Antimicrob Agents Chemother.

[5] Aquinas S, Allan W, Horsfall P (1972). Adverse reactions to daily and intermittent rifampicin regimens for pulmonary tuberculosis in Hong Kong. BMJ.

[6] Eule H, Werner E, Winsel K, Iwainsky H (1974). Intermittent chemotherapy of pulmonary tuberculosis using rifampicin and isoniazid for primary treatment: the influence of various factors on the frequency of side-effects. Tubercle.

[7] Martínez E, Collazos J, Mayo J (1999). Hypersensitivity reactions to rifampin. Pathogenetic mechanisms, clinical manifestations, management strategies, and review of the anaphylactic-like reactions. Medicine (Baltimore).

[8] Zierski M (1976). Adverse reactions under intermittent rifampicin regimens. Pharmacology of Antibiotics.

[9] Grosset J, Leventis S (1983). Adverse effects of rifampin. Rev Infect Dis.

[10] Poole G, Stradling P, Worlledge S (1971). Potentially serious side effects of high-dose twice-weekly rifampicin. BMJ.

[11] Pujet J, Homberg J, Decroix G (1974). Sensitivity to rifampicin: incidence, mechanism, and prevention. BMJ.

[12] Worlledge S (1976). Rifampicin-induced antibodies. Pharmacology of Antibiotics.

[13] Girling DJ, Hitze KL (1979). Adverse reactions to rifampicin. Bull World Health Organ.

